# The availability of health information to patients with newly diagnosed polymyalgia rheumatica: results from the Polymyalgia Rheumatica (PMR) Cohort study

**DOI:** 10.1017/S1463423618000543

**Published:** 2018-08-08

**Authors:** Maatla Tshimologo, Toby Helliwell, Samantha Hider, Christian Mallen, Sara Muller

**Affiliations:** 1 Medical Student, Research Institute for Primary Care & Health Sciences, Keele University, Keele, UK; 2 NIHR Clinical Lecturer, General Practitioner, Research Institute for Primary Care & Health Sciences, Keele University, Keele, UK; 3 Reader in Rheumatology, Consultant Rheumatologist, Research Institute for Primary Care & Health Sciences, Keele University, Keele, UK; 4 Haywood Academic Rheumatology Centre, Haywood Hospital, Stoke-on-Trent, UK; 5 NIHR Professor of General Practice Research, Research Institute for Primary Care & Health Sciences, Keele University, Keele, UK; 6 Senior Research Fellow in Epidemiology and Applied Statistics, Research Institute for Primary Care & Health Sciences, Keele University, Keele, UK

**Keywords:** glucocorticoids, information, patient and public involvement, polymyalgia rheumatica, survey

## Abstract

**Aim:**

The aim of this study was to assess the provision of information to, and seeking of information by, patients newly diagnosed with polymyalgia rheumatica (PMR) in primary care.

**Background:**

PMR is an inflammatory rheumatological condition of older people that can be treated with long-term oral glucocorticoids. Management usually requires the patient to understand the potential complications of treatment and the disease, as well as involvement in reducing treatment dose. This may be complex for patients to understand.

**Method:**

Data are taken from the baseline phase of the PMR Cohort study, which recruited newly diagnosed patients with PMR from UK primary care. Participants provided information on their PMR symptoms, general health and sociodemographics. They also completed items regarding information provision by their doctor at diagnosis, its usefulness and their own search for information.

**Findings:**

A total of 652 people responded to the baseline survey. In all, 399 (62.7%) had received written information from their doctor; 237 (98%) found it useful; 265 (42.9%) would have liked more information; and 311 (48.4%) sought out more information. Those who were not given information and did not seek it out tended to be older and have poorer internet access.

Information provided at diagnosis to patients with PMR is useful, but more than a third did not receive any. This is concerning when PMR requires self-management and vigilance for red flags. Doctors should make use of the resources already available to them to support patients and should specifically ensure that these are available to more elderly patients and those without internet access.

## Introduction

Polymyalgia rheumatica (PMR) is a chronic inflammatory musculoskeletal condition of unknown aetiology (González-Gay *et al*., 2017). It is more prevalent in people aged over 50 years and affects more women than men. Typical presenting symptoms include symmetrical pain and stiffness of the shoulder girdles and hips (Mackie, 2013), which have a significant impact on patients’ health, function and well-being (Helliwell *et al*., 2016). PMR is also closely associated with the condition giant cell arteritis (GCA), with up to one in five patients with PMR being diagnosed with GCA. GCA is a large-vessel vasculitis and, if left untreated, can result in irreversible visual loss. As such, patients need to be aware to consult early with symptoms such as new-onset headache or visual disturbance to reduce the risk of visual loss.

The majority of PMR patients are managed in primary care where they receive treatment with glucocorticoids, which are usually reduced slowly and discontinued after around two years. Owing to potential adverse effects related to glucocorticoid treatment, additional therapies to prevent these side effects (such as bisphosphonates for osteoporosis prevention and proton pump inhibitors for gastric protection) are also advised (Hernández-Díaz and Rodríguez, 2001; Dasgupta *et al*., 2010).

Therefore, vigilance for associated red flag symptoms, as well as the intricacies of a new treatment regimen with potential adverse effects, results in a significant increase in the complexity of self-care for newly diagnosed PMR patients. The chronic nature of PMR, the polypharmacy that PMR brings and the need to be aware of potential complications such as GCA means that these patients with PMR need to be well informed about their condition and its treatment to facilitate successful self-management and prevent potential complications. This is essential if they are to remain healthy, active, maintain function, identify disease associations (like GCA) and reduce potential complications. Ensuring successful self-management requires adequate provision of relevant information and an understanding of the condition. A large-scale systematic review of health literacy interventions across a range of conditions showed that a lack of understanding was associated with poorer physical and mental health, frequent consultations and overall reduced quality of life (Berkman *et al*., 2011). Furthermore, our work with patient stakeholders suggested that access to information was a priority for research.

It might be expected that doctors first provide patients with information on their condition at the time of initial diagnosis. This can be verbal, although increasingly it is recommended that clinicians supplement this by providing written information, such as information leaflets from NHS bodies and charities. To date, there are no data on the provision of information to patients with PMR from healthcare professionals or on what resources patients may use themselves. Information provided by doctors can be more disease and treatment orientated, and patients with chronic conditions may access internet forums and specific-disease online patient groups to learn more about their condition and how their condition can be more effectively self-managed (Li *et al*., 2014; Solberg, 2014).

Bearing in mind the wealth of publicly available information and the concerns of patient stakeholders regarding access to high-quality information, this study aimed to investigate written information provision for PMR patients at the time of their diagnosis and whether their information needs were met. Specifically, we consider that certain sociodemographic groups were not given information and whether there were groups who were not given information and did not access it themselves and therefore may have less information about their disease and its consequences.

## Methods

### Data collection

This study uses data from the PMR Cohort study. This is a primary care inception cohort of patients with PMR referred from 382 recruiting general practices between 2012 and 2014. The primary aim of the study was to consider the natural history and prognosis of PMR in primary care. A total of 652 patients participated in this study. The methods of the study have been presented elsewhere (Muller *et al*., 2012; 2016), but briefly newly diagnosed PMR patients (according to the British Society of Rheumatology guidelines) were referred into the study by their GP. Patients were mailed a baseline questionnaire, which included symptom severity and current treatment, sociodemographics and patients’ information needs for their condition.

We assessed the socioeconomic status of the participants according to their current employment status [employed; retired; other (including unemployed, sick, housewife)] and occupational class (higher managerial, administrative and professional; intermediate; routine and manual) (Office for National Statistics, 2010).

Information needs about PMR were assessed using the following questions: (1) Did the doctor give you written information about PMR? (yes/no) (2) If so, did you find it useful? (yes/no) (3) Have you looked elsewhere for information (eg, contacted a charity, searched on the internet?) (4) Do you have access to the internet as and when you want it? (yes/no).

In addition, we combined items 1 and 3 to define those who had and did not have access to any PMR information (information: GP gave written information and/or looked elsewhere for information; no information: GP did not give written information and participant did not look elsewhere for information).

Items were developed specifically for this study and based on the format of previously validated items (eg, Krumholz HM *et al*., *Circulation*. 1998; 97: 958–996). All draft study materials were reviewed and revised by patient stakeholders in order to ensure that they were accessible and comprehensible to the study sample. Patients were followed up by means of a questionnaire over two years. This paper presents data from the baseline phase only.

Ethical approval for the study was received from the Staffordshire Research Ethics Committee (REC reference number: 12/WM/0021), and all patients provided written informed consent.

### Statistical analysis

Percentages and means (standard deviations) were used to describe the sample and the information provided to them. The *χ* ^2^ and *t*-tests were performed as appropriate to assess the association between information provision and sociodemographic characteristics and access to the internet.

All analyses were conducted in Stata 14.2 (StataCorp, 2015) and IBM SPSS Statistics 24 (IBM Corp, 2016).

## Results

A total of 652 people (90.1%) completed the baseline questionnaire. In all, 405 (62.1%) were female and the mean (standard deviation) age of the respondents was 72.4 years (9.3) (Table 1). The majority of the sample was retired (*n*=512, 79.4%), with 76 (11.8%) still in employment. There was an even distribution of participants across socioeconomic groups. We have previously shown that those who did and those who did not respond to the baseline questionnaire were similar in terms of their age, gender and levels of neighbourhood deprivation (Muller *et al*., 2016). A total of 398 people (62%) reported having access to the internet when they wanted it. This was slightly higher in males than in females, but there was no statistically significant difference (64.9 versus 60.1%; *P*=0.227).

**Table 1:** Characteristics of the cohort sample [*n* (%) unless otherwise stated]

	Female	Male	All
Female gender	405 (62.1)	247 (37.9)	652 (100)
Age (mean, SD)	71.8 (10.0)	73.3 (7.8)	72.4 (9.3)
Current employment status			
Employed	47 (11.7)	29 (11.9)	76 (11.8)
Retired	309 (77.1)	203 (83.2)	512 (79.4)
Other	45 (11.2)	12 (4.9)	57 (8.8)
Occupational class			
Higher managerial, administrative, professional	76 (27.7)	68 (39.5)	144 (32.3)
Intermediate	85 (31.0)	40 (23.3)	125 (28.0)
Routine and manual	113 (41.2)	64 (37.2)	177 (39.7)
Have internet access	241 (60.1)	157 (64.9)	398 (61.9)
Given information from the doctor	245 (61.9)	154 (64.2)	399 (62.7)
Information from the doctor was useful^a^	237 (98.3)	149 (97.4)	386 (98.0)
Would have liked more information	174 (45.7)	91 (38.4)	265 (42.9)
Looked elsewhere for information	200 (50.1)	111 (45.7)	311 (48.4)

a This is a proportion of those who got information from the doctor.

In total, 399 (62.7%) respondents reported receiving written information provided by their doctor about their PMR. There were no significant differences in the receipt of information in terms of age, gender, employment status, socioeconomic class or access to the internet (Table 2). In those who reported receiving written information from their doctor, the majority of individuals (237, 98%) found it useful. However, even in the group receiving written information, one in four patients (28.1%, *n*=109) would have liked more information from the doctor and 39.5% (*n*=157) reported having looked for further information elsewhere.

**Table 2:** Associations with having received written information from the doctor

	Received information from GP	Did not receive information from GP	*χ* ^2^/*t*-value; *P*-value
Age [mean (SD)]	72.1 (9.8)	72.2 (8.9)	−0.1646; 0.869
Male gender	154 (38.6)	86 (36.9)	0.3375; 0.561
Current employment status			1.6914; 0.429
Retired	308 (78.4)	189 (80.1)	
Employed	45 (11.5)	30 (12.7)	
Other	40 (10.2)	17 (7.2)	
Occupational class			4.9714; 0.083
Routine and manual	119 (42.8)	53 (33.1)	
Intermediate	78 (28.1)	46 (28.8)	
Higher managerial, administrative, professional	81 (29.1)	61 (38.1)	
Access to internet	239 (59.9)	156 (66.4)	2.6468; 0.104

In those who did not receive information from their doctor, 67.9% (*n*=152) would have liked more information and 63.6% (*n*=150) looked for further information. However, there was no association between wanting more information from the doctor and patients looking for additional information themselves (*P*=0.366).

A total of 86 individuals (13.5%) reported neither receiving written information from their doctor nor looking for information themselves. This group could be thought of as having not received any written information on PMR. These people were on average older (75.7 versus 71.7 years; *P*=0.0001) than those who received information, and fewer had internet access (28.2 versus 67.6%; *P*<0.0001) (Table 3). However, there was no difference in terms of gender, employment status or occupational class (or most recent occupational class among those retired). In this group who did not receive any information, 71.6% (*n*=58) would have liked more information from their GP.

**Table 3:** Characteristics of those with and without polymyalgia rheumatica information (from GP or found themselves)

	Information	No information	*χ* ^2^/*t*-value; *P*-value
Female gender	349 (63.1)	49 (57.0)	1.1919; 0.275
Age (mean, SD)	71.7 (9.3)	75.7 (7.9)	3.8205; 0.0001
Current employment status			4.8295; 0.089
Employed	71 (13.0)	4 (4.7)	
Retired	427 (78.1)	73 (85.9)	
Other	49 (9.0)	8 (9.4)	
Occupational class			0.5262; 0.769
Higher managerial, administrative, professional	122 (32.0)	21 (36.8)	
Intermediate	109 (28.6)	15 (26.3)	
Routine and manual	150 (39.4)	21 (36.8)	
Have internet access	374 (67.6)	24 (28.2)	48.7271; <0.0001

## Discussion

The chronicity of PMR and potential glucocorticoid complications means that patients need to be able to understand the condition and its treatment to successfully facilitate self-management. Provision of adequate health information is key to patient understanding of health conditions, but has never previously been considered in PMR. In this PMR inception cohort, less than two-thirds of participants reported being given written information about PMR by their doctor. Although nearly all of those who were given information found it useful, over a quarter of these people would have liked more information and nearly two in five looked for other sources of additional information. Over two-thirds of people who did not receive information from their doctor would have liked to have received information.

More than one in eight participants reported not being given or looking for information on PMR around the time of their diagnosis. These people were on average older and fewer had access to the internet than people who received information either from their GP or by finding it themselves. The lack of association between age and provision of information by the doctor suggests that this is related to internet access in older people (Office for National Statistics, 2017). Regardless of the reason for the lack of information in this group, they represent a missed opportunity to provide information and potentially improve self-management, as over 70% reported that they would have liked more written information from their doctor. Although previous authors have suggested that giving information to patients who did not want to receive it may diminish the chance of improving outcomes (van Weel-Baumgarten, 2008), our data suggest otherwise. We would therefore encourage health care professionals to furnish patients with the information that they need to manage their PMR and that our patient stakeholders, as well as research participants, suggest that they want.

Our findings are in agreement with previous studies on rheumatoid arthritis and ankylosing spondylitis where patients report general satisfaction with the written information they were given by doctors (Rosemann *et al*., 2006). Previous research has shown that people from higher occupational classes tended to have higher information expectations and were usually not satisfied with information they received (Stark *et al*., 2014), although this was not confirmed by our data.

A previous study in patients with rheumatoid arthritis has suggested that because of the evolving nature of the condition, there is no such thing as a ‘fully informed patient’ (Kjeken *et al*., 2006). This may be the case, to a lesser extent, in PMR and patients may never receive all of the information they need, as what is relevant to them will change over time and be different for different people. Information on the general medical aspects of PMR is easily available on the Arthritis Research UK website and is also available from GP leaflets on PMR. However, online information has previously been shown to have poor readability characteristics for older adults (Vivekanantham *et al*., 2017).

The majority of patients diagnosed with PMR have never previously heard of this condition (Muller, In press), and it is known that receiving a diagnosis of a condition that you were previously unaware of makes it harder to digest information, as you come to terms with a new diagnosis (McClain and Buchman, 2012). This further reiterates the importance of the provision of written information to these patients.

This nationwide, primary care-based study has a large sample size and excellent response rate, and the items described in this article were developed in association with patient stakeholders. The choice of primary care setting reflects practice in the United Kingdom, where <20% of those with a diagnosis of PMR ever see a rheumatologist for the condition (Barraclough *et al*., 2008). However, the demographic characteristics of the sample are typical of those recruited from specialist settings in other studies. This gives confidence in the diagnosis of these patients while reducing the potential for spectrum bias (ie, a more severe or atypical group of patients than would be recruited in a specialist setting). The completion of the baseline questionnaire in the week following diagnosis will also limit the possibility for recall bias, although it also limits the time in which patients have had the opportunity to conduct their own research into PMR and to search out further information. As GPs were asked to recruit patients into this study at the time they made their diagnosis of PMR, it is possible that this process of study inclusion altered the usual information provision by the GP either because they were triggered to consider the need for information or conversely that time pressures meant they neglected to provide information that they usually would. However, we expect any such phenomenon to have a negligible effect on our results.

Finally, we do not know what information GPs provided to patients or what information patients might have liked to receive, or indeed what information was accessed by those who looked for it themselves. Many GP computer systems in the United Kingdom provide links to NHS-supported health information websites, such as patient.co.uk. It is therefore likely that the information that patients received was printed from these sites. In addition, some practices may also have had access to leaflets from charities such as Arthritis Research UK, or GPs may have signposted patients to charities, their websites or patient support groups (eg, PMRGCAuk). We do, however, know that the information that was provided was found to be helpful by patients. This suggests that current sources of information are useful and could be provided to all patients. What is less clear is what information was found by patients who looked for it themselves and what potential consequence this could have (eg, for treatment adherence).

Although the current study has provided the first data regarding the provision of written health information to patients newly diagnosed with PMR and we know that those who received information were satisfied with it, further studies need to investigate the quality of information provision. A previous study showed that information on the internet regarding PMR was often written in too complex a form to be accessible to the average older person (Vivekanantham *et al*., 2017). It would also be beneficial to further our understanding of the formats in which patients would like to receive information (eg, leaflets, DVD, online text/video clip). To fully realise the benefits of health information supplied in PMR, and other conditions, it may be necessary to evaluate the understanding gained by patients from their use.

The reasons for information not being provided to patients by doctors are unclear, as we were unable to identify any sociodemographic factors associated with the provision of information. The group who did not access information via the GP or their own research were older and had less access to the internet; these individuals did not look for the information themselves. This suggests that those who are older and the GP knows or suspects do not have good internet access should be targeted to receive information from their GP as they are less likely to seek information themselves.

## Conclusion

The provision of information to patients with PMR is important, as its management can be complex and requires a collaborative doctor–patient relationship. Doctors diagnosing and treating PMR should make use of the resources already available to them to support patients and should specifically ensure that these are available to their more elderly patients and those who cannot access the information themselves via the internet.
